# Efficient extraction and antioxidant activity of polyphenols from *Antrodia cinnamomea*

**DOI:** 10.1186/s12896-022-00739-5

**Published:** 2022-03-07

**Authors:** JianZhi Chu, YongFei Ming, Qi Cui, Na Zheng, ShuDe Yang, WeiHuan Li, Hongwei Gao, Rui Zhang, XianHao Cheng

**Affiliations:** 1 Shandong Key Lab of Edible Mushroom Technology, School of Agriculture, Ludong University, Yantai, 264025 China; 2 School of Life Science, Ludong University, Yantai, 264025 Shandong China

**Keywords:** *Antrodia cinnamomea*, Polyphenols, Antioxidant, Extraction, Deep eutectic solvents (DESs)

## Abstract

**Background:**

*Antrodia cinnamomea*, a rare medicinal fungus, has been increasingly studied in recent years because of its abundant secondary metabolites which are beneficial to humans. However, there is a lack of research on its polyphenols which are of good research value due to their antioxidant, anti-inflammatory, hypoglycemic and other activities.

**Results:**

In this study, the effects of different extraction conditions on the yield of its polyphenols were investigated. Deep-Eutectic Solvents composed of choline chloride and malonic acid had the best extraction efficiency, with the optimal extraction conditions being as follows: a solid–liquid ratio of 40 mg/mL, an extraction temperature of 55 °C, an extraction time of 70 min and a DES with 20% water content. Under these conditions, the extraction yield of polyphenols reached 22.09 mg/g which was about 2 times that of alcohol-based extraction (10.95 mg/g). In vitro antioxidant test results further showed that polyphenols from *A. cinnamomea* had strong antioxidant activities. When the concentration of polyphenols reached 0.1 mg/mL of polyphenols, the scavenging activity of free radical basically reached its maximum, with values of 94.10%, 83.34% and 95.42% for DPPH, ABTS^+^ and ·OH scavenging. In this case, the corresponding IC_50_ values were 0.01, 0.014 and 0.007 mg/mL, respectively.

**Conclusions:**

This study lays the foundation for the efficient extraction and application of polyphenols from *A. cinnamomea.*

## Background

*Antrodia cinnamomea* (T. T. Chang & W. N. Chou 1995) belongs to the division *Basidiomycota* (R.T. Moore 1980), order *Polyporales* (Gäum. 1926), family *Fomitopsidaceae* (Jülich 1982) and genus *Antrodia* (P. Karst. 1879) [1]. It is a well-known but rare edible and medicinal fungus, mainly distributed within the Taiwan province of China where it is also known by the local people as “Niu-Chang-Chih” or the "Ruby of forest". It was first used in folk medicine when indigenous people in Taiwan used it as a cure for liver problems caused by alcohol intoxication based on the belief that its regular consumption could keep the body active while prolonging life. Although *A. cinnamomea* exists since a long time, a formal documentary report was not published until 1990. In the 1990s, Zang Mu, a scholar in Kunming, mistook the sample as *Ganoderma* (P. Karst. 1881), and named it *Ganoderma camphoratum* (M. Zang & C. H. Shu 1990) [2]. In 1995, Tun-Tschu Chang, a researcher from Taiwan, named *A. cinnamomea* and published it as a new species based on its morphological characteristics [3]. In 1997, Wu Shenghua found that *G. comphoratum* and *A. cinnamomea* were the same species, and therefore, this fungus was given the new name *Antrodia camphorata* (Sheng H. Wu, Ryvarden & T. T. Chang) [4]. Nowadays, this mushroom is known as *A. cinnamomea*, although the name *A. camphorata* is also used. Wild *A. cinnamomea* has the aroma of a Bull Camphor tree, along with a bitter taste, an irregular shape as well as a fruiting body without stipe. At first, the fruiting body is of orange red and orange brown colour, but as time goes by, it becomes brown or blackish [3]. The Bull Camphor tree is the only host for wild *A. cinnamomea* and only grows in the mountains at 450–1200 m above sea level in Taiwan [3], while *A. cinnamomea* only grows slowly on the inner wall of decayed Bull Camphor tree. The number of Bull Camphor trees has declined sharply in recent years due to excessive felling of the tree, which is now listed as a protected species. Since wild Bull Camphor tree are rare and valuable, artificial rearing of *A. cinnamomea* are beginning to enter the market. Due to the scarcity of wood resources in the section of Bull Camphor tree, researchers mostly use other sections to cultivate and produce fruit bodies of Bull Camphor tree, such as other camphor tree, the old apple wood, pear wood, chestnut wood and oak wood [5, 6]. In addition to cut-log cultivation, liquid state fermentation and solid-state fermentation are also common cultivation methods. These two methods of culture greatly shorten the growth cycle of *A. cinnamomea*, and can be produced on a large scale, which attracts the attention of researchers.

A number of active substances, including terpenoids, polysaccharides, ubiquinone derivatives (antroquinonol), maleic and succinic acid derivatives as well as polyphenols, just to name a few, can be found in *A. cinnamomea*. At present, studies involving this fungus are mainly focused on antcins, antroquinonol and polysaccharides [7–15]. On the other hand, only few are based on polyphenols despite the fact that modern pharmacological studies have shown that these compounds have significant hypoglycemic, anti-tumor, liver protection, antioxidant and anti-inflammatory effects [16–18]. Therefore, research on the extraction and pharmacological activity of polyphenols from *A. cinnamomea* has a broad prospect. Current extraction methods of polyphenols is based on organic solvent extraction [19], ultrasound-assisted extraction [20], microwave-assisted extraction [21], supercritical fluid extraction [22] and enzyme aided extraction [23] and among these, the organic solvent extraction is the most widely used. However, these traditional extraction methods have a number of disadvantages in terms of long extraction time, high extraction cost, low extraction efficiency, environmental pollution, complex process and low purity of extract. Therefore, it is very important to find alternative green solvents with high efficiency, low cost along with little to no pollution.

In this context, Deep-Eutectic Solvents (DESs) which were first proposed by Abbot and his team [24], represent a new and green group of extraction solvents. DESs are usually formed by interactions between two or three compounds through hydrogen bonds. In this case, hydrogen bonds can be formed between the hydrogen bond donor and the hydrogen bond acceptors which are generally quaternary ammonium salts such as quaternary ammonium halide anions. These interactions lead to the delocalization of charge, eventually resulting in the melting point of the mixture being lower than that of each component of the solvent [25]. These solvents also have many advantages, such as lower prices, easy preparation as well as easy storage while being environment-friendly, with these benefits attracting increasing attention of researchers. Hence, DESs have found applications as reaction media in biocatalysis, synthesis of organic compounds, and extraction of natural active substances [26–28]. In this paper, using choline chloride as the hydrogen bond acceptor, eight types of DESs were prepared by adding different hydrogen bond donors in a certain proportion in order to screen for DES with the highest extraction efficiency of polyphenols from *A. cinnamomea*. The optimal extraction conditions were explored by single factor experiments. On this basis, the extraction process was optimized by response surface design. In addition, in vitro antioxidant activities of the extracted polyphenols were also determined. It is expected that this study will lay the foundation for further studies on the pharmacological activity of polyphenols in *A. cinnamomea* for the development of drugs.

## Results

### Effects of the water content of ethanol on the extraction yield of polyphenols from *A. cinnamomea*

**Fig. 1:**
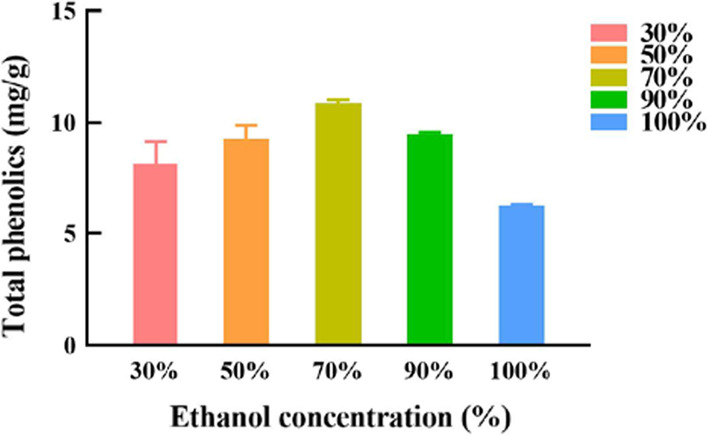
The effect of ethanol concentration on the extraction yield of polyphenols in *A. cinnamomea*

Ethanol of different concentrations was also used as the control to reflect the advantages of DES. The experimental results, shown in Fig. 1, indicated that when 70% ethanol was used as the extraction solvent, the extraction yield reached 10.95 mg/g.

### Effects of different DESs on the extraction yield of polyphenols from *A. cinnamomea*

**Fig. 2:**
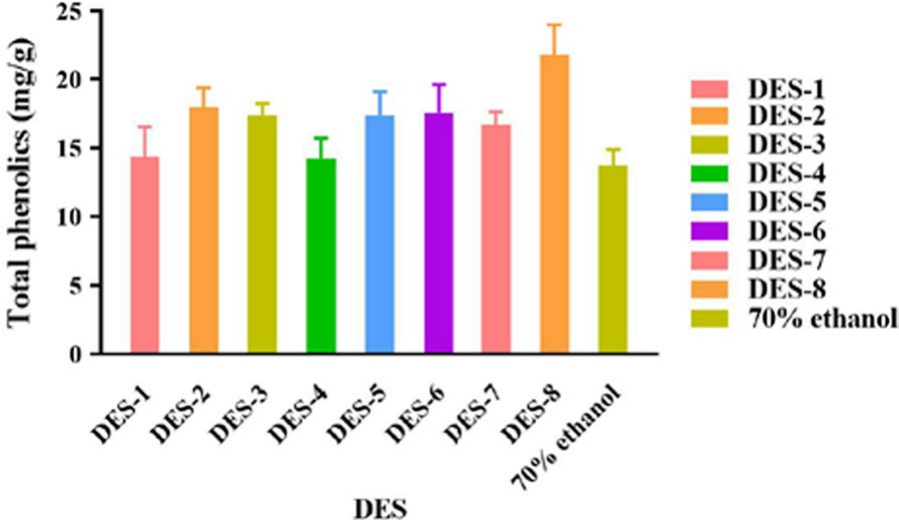
The effect of different deep eutectic solvents on the extraction yield of polyphenols in *A. cinnamomea*

Differences in the type of hydrogen donors used for preparing various formulations of DESs resulted in variations in the extraction yield of polyphenols from *A. cinnamomea*. Eight different DESs with 40% water content were used to extract polyphenols from *A. cinnamomea* using a starting solid–liquid of 50 mg/mL and 70% ethanol as the control. The results were as shown in Fig. 2. The amount of polyphenols extracted from *A. cinnamomea* was higher than that from 70% ethanol with each of the eight different DESs. Of these, the highest yield of polyphenols was 21.83 mg/g, obtained with the DES composed of choline chloride and malonic acid (DES-8). In fact, the extraction efficiency of polyphenols by this DES was 1.68 times that of 70% ethanol and it was, therefore, selected as the extraction solvent for the subsequent single factor test. The results also showed that DES have great potential for the extraction of polyphenols.

### Results from single factor experiments

#### Effects of the ratio of the material liquid on the extraction yield of polyphenols

**Fig. 3:**
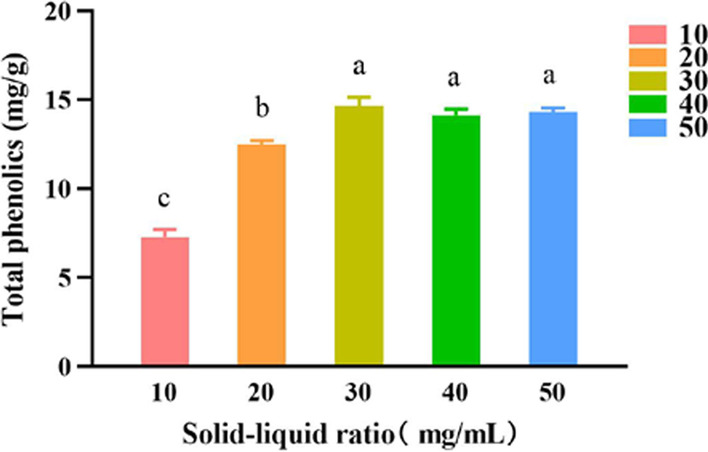
Effects of different ratio of the material liquid on the extraction yield of polyphenols of *A. cinnamomea.* SPSS analysis showed that differences were between groups 30 and 20, 30 and 10, 40 and 20, 40 and 10, 50 and 20, 50 and 10, 20 and 10 (*P* < 0.05), and are marked by the different letters (e.g., 10, 20, 30)

The experimental results, shown in Fig. 3, indicated that when the solid–liquid was in the range of 10–50 mg/mL, the extraction efficiency of polyphenols initially increased before becoming almost constant. SPSS analysis, in this case, demonstrated that the extraction yields were significantly different from each another (*P* < 0.05). Taking into account the economic cost associated with high concentrations, 30 mg/mL was selected as the best one for the material.

#### Effects of the extraction temperature on the extraction yield of polyphenols

**Fig. 4:**
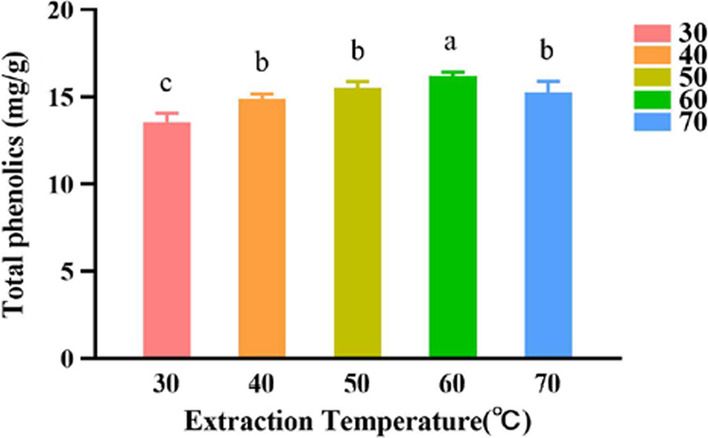
Effect of different extraction temperatures on the extraction yield of polyphenols of *A. cinnamomea.* SPSS analysis showed that differences were between groups 60 and 40, 60 and 50, 60 and 70, 60 and 30, 40 and 30, 50 and 30, 70 and 30 (*P* < 0.05), and are marked by different letters

Studies have shown that high temperatures can promote the formation of low viscosity and surface tension DES [25]. The experimental results (Fig. 4) showed that with increasing temperature, the extraction yield of polyphenols increased to reach its maximum at 60 °C. The yield then decreased at higher temperatures. Overall, the five temperatures produced significantly different yields (*P* < 0.05). Therefore, 60 °C was chosen as the optimal extraction temperature.

#### Effects of the extraction time on the extraction yield of polyphenols

**Fig. 5:**
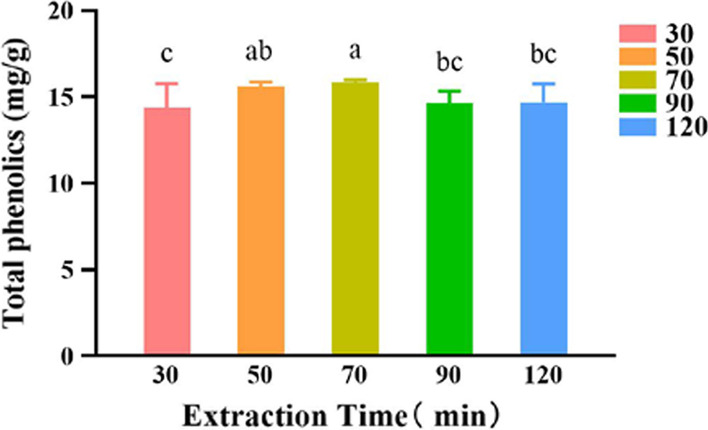
The effect of different extraction time on the extraction yield of polyphenols of *A. cinnamomea.* SPSS analysis showed that differences were between groups 70 and 30, 70 and 90, 70 and 120, 50 and 30 (*P* < 0.05), and are marked by different letters (e.g., 30, 70)

The experimental results are shown in Fig. 5. With increasing extraction time, the extraction yield of polyphenols gradually increased, reaching the maximum at 70 min, before subsequently displaying a slight decrease. Statistical analysis further demonstrated that the five extraction times yielded significantly different amounts of polyphenols (*P* < 0.05). Therefore, 70 min was selected as the best extraction time.

#### Effects of the water content of DES on the extraction yield of polyphenols

**Fig. 6:**
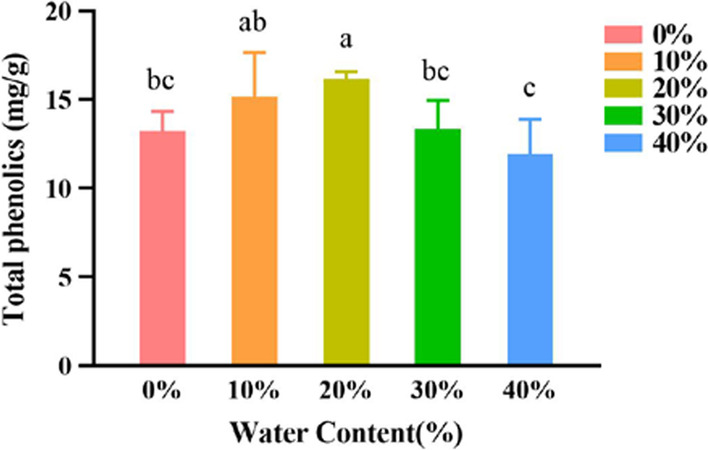
Effect of different water content of deep eutectic solvent on the extraction yield of polyphenols of *A. cinnamomea.* SPSS analysis showed that differences were between groups 20% and 0%, 20% and 30%, 20% and 40%, 10% and 40% (*P* < 0.05), and are marked by the different (e.g., 20%, 40%)

The experimental results were as shown in Fig. 6. The extraction yields of polyphenols were different for DES-8 with different moisture content. When the water content was 20%, the extraction yield of polyphenols reached its maximum of 16.16 mg/g and SPSS analysis showed that the yield of polyphenols were significantly different for the five percentage of water content (*P* < 0.05). The optimal moisture content was, therefore, determined to be 20%.

### Optimization of the polyphenol extraction process by response surface methodology

The Design-Expert10 software design scheme as well as the results of the experiment are shown in Table 1. Multiple linear regression analysis was then conducted based on the results, with the linear regression equation of the experiment being as follows:

1 $$\begin{aligned} {\text{y}} & = {21}.{56} + {3}.{\text{41A}} - 0.{\text{82B}} - 0.{\text{78C}} + 0.0{\text{79D}} - 0.{\text{51AB}} \\ & \quad + 0.{\text{23AC}} - 0.0{\text{18AD}} - 0.0{5}0{\text{BC}} - 0.{\text{32BD}} \\ & \quad - 0.{\text{11CD}} - {4}.{\text{13A}}^{{2}} - {3}.{8}0{\text{B}}^{{2}} - {2}.{3}0{\text{C}}^{{2}} - {1}.{\text{27D}}^{{2}} \\ \end{aligned}$$

**Table 1:** Response surface experimental design scheme and results

Number	Ratio of the material liquid (mg/mL) A	The water content (%) B	Extraction temperature (°C) C	Extraction time (min) D	Polyphenols content (mg/g)
1	10	20	40	70	12.48
2	30	20	80	40	17.43
3	30	20	40	40	18.67
4	50	0	60	70	17.67
5	30	20	60	70	21.18
6	30	40	60	100	15.27
7	30	0	80	70	15.73
8	50	20	60	100	19.68
9	30	0	60	100	17.87
10	10	0	60	70	10.51
11	30	20	40	100	18.89
12	50	20	40	70	19.45
13	10	20	60	40	12.48
14	10	20	60	100	12.84
15	30	0	60	40	17.08
16	50	20	60	40	19.40
17	30	40	40	70	15.16
18	30	20	80	100	17.20
19	30	40	80	70	13.52
20	10	20	80	70	10.35
21	50	40	60	70	15.83
22	30	20	60	70	21.42
23	30	20	60	70	22.36
24	30	40	60	40	15.74
25	30	20	60	70	22.13
26	10	40	60	70	10.71
27	30	0	40	70	17.17
28	50	20	80	70	18.24
29	30	20	60	70	20.71

Results of the regression equation are shown in Table 2. From the equation, the correlation coefficient was determined to be R^2^ = 0.9888. For the regression model, the *P*-value was < 0.0001 and was, therefore, significant. At the same time, the lack of fit value was 0.88 (*P* > 0.05), hence indicating that the selected model was not only reasonable and feasible, but could also be used to analyze or predict the extraction of polyphenols. Therefore, the above model can be used for the analysis and prediction of polyphenol content in *A. cinnamomea*. Based on the analyses, The *P* values of A, B and C were < 0.05, indicating that these three factors had significant effects on the extraction yield of polyphenols. At the same time, by comparing the F value, the influence of each factor on the extraction yield could be determined, with the influence of each factor likely to be A > B > C > D.

**Table 2:** Variance analysis of extraction yield regression model

Source model	Sum of squares	*df*	Mean square	F value	*P* value Prob > F	Significant
	336.18	14	24.01	88.22	< 0.0001	
A–A	139.35	1	139.35	511.96	< 0.0001	
B–B	8.01	1	8.01	29.44	< 0.0001	
C–C	7.28	1	7.28	26.76	0.0001	
D–D	0.074	1	0.07	0.27	0.60	
AB	1.04	1	1.04	3.82	0.07	
AC	0.21	1	0.21	0.78	0.39	
AD	1.34E-03	1	1.34E-03	4.93E-03	0.94	
BC	0.01	1	0.01	0.04	0.85	
BD	0.40	1	0.40	1.47	0.24	
CD	0.05	1	0.05	0.19	0.66	
A^2^	110.75	1	110.75	406.87	< 0.0001	
B^2^	93.84	1	93.84	344.75	< 0.0001	
C^2^	34.34	1	34.34	126.15	< 0.0001	
D^2^	10.43	1	10.43	38.30	< 0.0001	
Residual	3.81	14	0.27			
Lack of fit	1.96	10	0.20	0.42	0.88	Not significant
Pure error	1.86	4	0.46			
Cor total	339.99	28				
R^2^	0.9888					

**Fig. 7:**
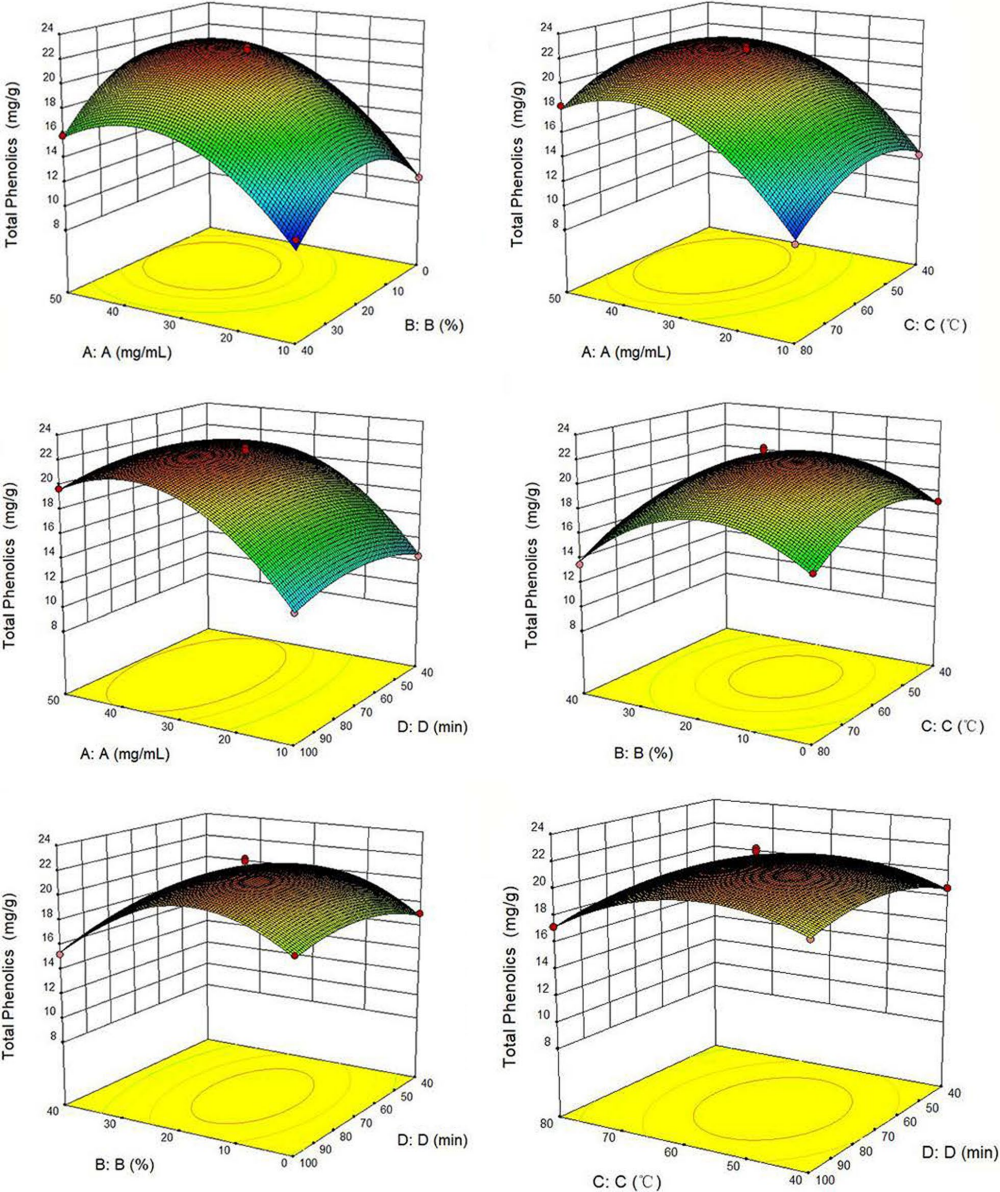
Response surface of the effects of interaction of various factors on the extraction yield of polyphenols of *A. cinnamomea*

A 3D response surface map was used to represent the interaction between various factors (Fig. 7). The surfaces for the ratio of the material liquid (A), water content (B) and extraction temperature (C) were relatively steep, while the change in the extraction time (D) was relatively gentle. Hence, the results showed that A, B and C had a great effect on the extraction yield of polyphenols, while D had a comparatively smaller effect.

Based on the results, the optimal extraction conditions were determined to be as follows: a concentration of the material of 39.12 mg/mL, a water content of 16.88% for DES-8, an extraction temperature of 56.03 °C, and an extraction time of 73.36 min. Using these optimal conditions, the predicted extraction yield of polyphenols was 22.36 mg/g. To ensure the feasibility of the experiment, the optimal extraction conditions were modified as follows: the concentration of the material was 40 mg/mL, the water content was 20% for DES-8, the extraction temperature was 55 °C, and the extraction time was 70 min. Based on these improved conditions, the amount of polyphenols extracted was 22.09 mg/g, with a relative deviation of 1.21%. Both values were consistent with the corresponding theoretical ones, thus indicating that the model was reasonable and feasible. Overall, this model can be used to predict and analyze the extraction yield of polyphenols from *A. cinnamomea.*

### Antioxidant Activity of polyphenols from *A. cinnamomea*

#### Results for the DPPH scavenging activity of polyphenols from *A. cinnamomea*

**Fig. 8:**
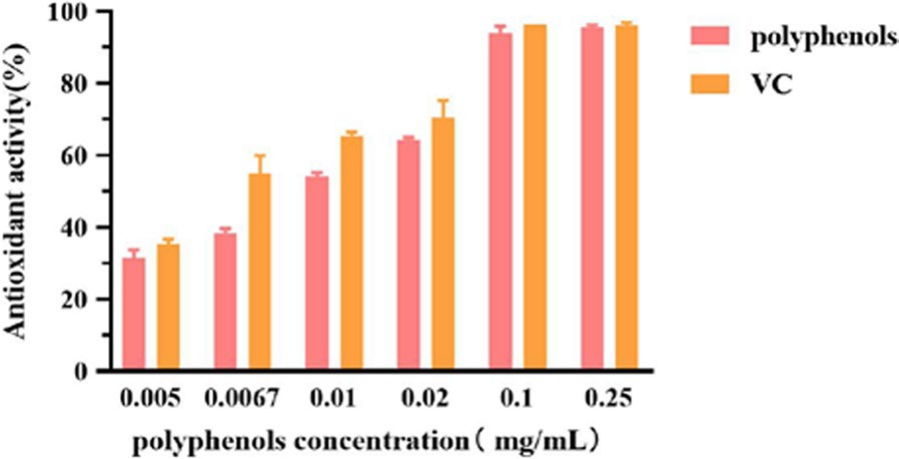
DPPH scavenging activity of polyphenols

The scavenging activity for DPPH is shown in Fig. 8. With VC as the control, the scavenging effect of polyphenols on DPPH was clearly comparable. Within the range of 0.005–0.25 mg/mL, the scavenging effect increased with increasing concentrations of polyphenols until it reached 0.1 mg/mL where the scavenging effect was the highest at 94.10%. Increasing the concentration of polyphenols beyond this point had little effect on the antioxidant activity. For this assay, the IC_50_ values for polyphenols and VC were 0.01 and 0.007 mg/mL, respectively. These results showed that it indicated that polyphenols in *A. cinnamomea* had a high scavenging ability on DPPH free radicals.

#### Results for ABTS^+^ scavenging activity of polyphenols from ***A. cinnamomea***

**Fig. 9:**
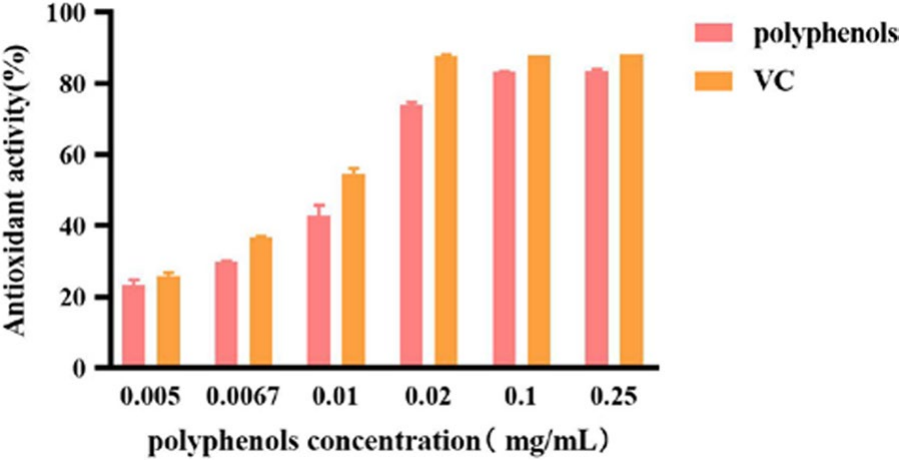
ABTS^+^ scavenging activity of polyphenols

Results for the ABTS^+^ scavenging activity (Fig. 9) showed that within the range of 0.005–0.25 mg/mL, the scavenging activity increased before becoming almost constant at higher concentrations of polyphenols. When the concentration reached 0.1 mg/mL, ABTS^+^ radical scavenging effects reached 83.34% (94.65% for that of VC). In this case, the IC_50_ values for polyphenols and VC were 0.014 and 0.009 mg/mL, respectively. Further increases in concentration had little effect on ABTS^+^ scavenging effect and therefore, polyphenols in *A. cinnamomea* were found to have strong scavenging effect on ABTS^+^.

#### Results for the ·OH scavenging activity of polyphenols from *A. cinnamomea*

**Fig. 10:**
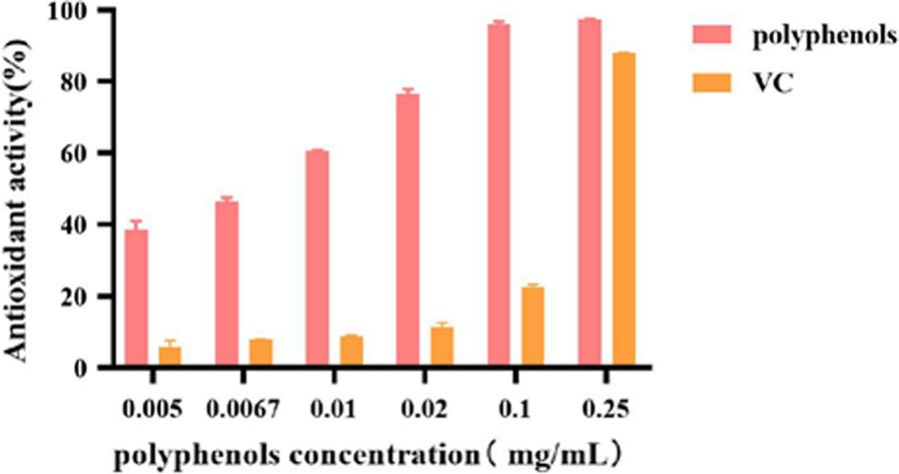
OH scavenging activity of polyphenols

Results for the scavenging effect on ·OH are shown in Fig. 10. Within the range of 0.005–0.25 mg/mL, the scavenging effect increased with increasing concentration of polyphenols. When the concentration reached 0.1 mg/mL, the scavenging effect reached 95.42%, with no obvious changes observed at higher concentrations. Based on the results, the IC_50_ values for polyphenols and VC were found to be 0.007 and 0.114 mg/mL, respectively. Overall, the polyphenols in *A. cinnamomea* had a much greater scavenging effect on ·OH than VC and hence, they have been shown to have good antioxidant activity in vitro.

## Discussion

So far, research on the active ingredients of *A. cinnamomea* has been mainly focused on triterpenoids, antroquinonol and polysaccharide compounds, while research on its polyphenols has been given less attention.

In this study, a new extraction method, involving DES, was used to extract polyphenols from *A. cinnamomea*. The use of DES greatly improved the extraction yield compared with the traditional alcohol extraction method but although DES can dissolve polyphenols more efficiently, different formulations actually have different extraction efficiency. In this study, eight different DESs were screened, and the experiment confirmed that DES-8, composed of choline chloride and malonic acid, was the most efficient. Previous studies have shown that *A. cinnamomea* are rich in highly polar active substances [33]. The polarity of DES-8 is higher than that of ethanol, and since the polyphenols have strong hydrogen bond interactions with the DES-8, they can be quickly dissolved in the DES during extraction. Therefore, DES-8 can be very efficient in the extraction of this type of compounds from *A. cinnamomea*. In the single factor experiment, when the water content of the deep eutectic solvent was low, the deep eutectic solvent had a certain level of viscosity which can hinder the interaction between the DES-8 and *A. cinnamomea*, thereby resulting in a low extraction yield. However, when the water content is too high, the concentration of DES-8 decreases, hence affecting the extraction efficiency of polyphenols. In addition, with increasing concentration of the material, the yield of polyphenols initially increased before becoming constant. This could be because increasing the solid to liquid ratio can cause the solubility of DES-8 for polyphenols to reach saturation. Temperature also had an effect on the extraction of polyphenols. With increases in temperature, the yield first increased before showing a subsequent decrease. This could be attributed to the increased solubility of *A. cinnamomea* mycelium powder in the solvent at higher temperatures. Furthermore, higher temperature could reduce the density and viscosity of deep eutectic solvent, thus improving interactions between the DES and polyphenols. However, at higher temperatures, it is also likely for part of the polyphenols to decompose, resulting in a decrease in the extraction efficiency. Finally, the extraction time had an effect on the yield of polyphenols. With increasing extraction time, the yield first increased before showing a slight decrease, which was probably caused by the structural changes in some polyphenols due to long ultrasonic time.

In terms of the antioxidant activity, the scavenging effects of the polyphenols on DPPH, ABTS^+^ and ·OH free radicals could reach more than 90% at low concentrations, thereby indicating that the polyphenols from *A. cinnamomea* can have good antioxidant activity.

The use of DES-8 in this experiment greatly increased the amount of polyphenols extracted from *A. cinnamomea*, but since the solvent was an acidic salt solution, compounds such as rutin, which are easily soluble in alkaline solution, might not be extracted [25]. Therefore, further studies are needed to successfully extract other types of polyphenols from *A. cinnamomea* using DES-8. In addition, there were also some limitations in these experiments. For example, the extracts used when determining the antioxidant activity were partially purified crude extracts as the purification process of polyphenols is complicated. This needs to be solved for future studies so that more cell and animal experiments can be conducted to better assess the pharmacological activities of polyphenols in *A. cinnamomea*.

## Conclusions

In general, this study laid a foundation for the efficient extraction and application of phenols from *A. Cinnamomea*. In this paper, a preliminary study on polyphenols from *A. cinnamomea* was carried out while exploring a new extraction method based on ultrasonic-assisted DESs extraction. With this method, the extraction yield of polyphenols was about two times higher than that obtained from traditional alcohol extraction. Therefore, DES extraction could be a better approach. By selecting different DES, it was also found that a combination of choline chloride and malonic acid (DES-8) was the best one for extracting polyphenols. Moreover, based on the single factor test and response surface analysis, the optimal conditions for ultrasonic-assisted deep eutectic solvent extraction were as follows: a water content of 20% for DES-8, a solid–liquid of 40 mg/mL, an extraction temperature of 55 °C, and an extraction time of 70 min. In vitro antioxidant test results further showed that the scavenging effects of the polyphenols on DPPH, ABTS^+^ and ·OH first increased in the concentration range of 0.005–0.25 mg/mL before becoming almost constant. When the concentration was 0.1 mg/mL, the scavenging activities were the highest, with values for DPPH, ABTS^+^ and ·OH assays being 94.10%, 83.34% and 95.42%, respectively. In particular, the polyphenols displayed good ·OH scavenging activity with an IC_50_ value of 0.007 mg/mL. The results indicated that the polyphenols of *A. cinnamomea* had strong antioxidant activity and could be used in drug as well as other health product development in the future.

## Materials and methods

### Materials, reagents, and instruments

Materials: *A. cinnamomea* was cultured for two months in our laboratory. The mycelium was dried at 45 °C, ground in a grinder, passed through a 40-mesh sieve (The aperture was 0.425 nm), and eventually stored at 4 °C until further use.

Reagents: Choline chloride, glucose, lactic acid, glycerol, 1,4-butanediol, malic acid, urea, malonic acid, sodium carbonate, potassium persulfate (K_2_S_2_O_8_), salicylic acid, ferrous sulfate as well as vitamin C (VC) were all of analytical grade and were obtained from Sinopill Chemical Reagents Co., Ltd. (Tianjin, China). Gallic acid monohydrate standards (purity > 98%) of analytical grade was purchased from Cool Chemical Technology Co., Ltd. (Beijing, China). Folin was obtained from Shanghai Jinsui Biotechnology Co., Ltd. (Shanghai, China) and analytical grade hydrogen peroxide (H_2_O_2_) was obtained from Tianjin Ruijin Chemical Co., Ltd. (Tianjin, China). 1,1-diphenyl-2-picrylhydrazyl (DPPH), 3-ethylbenzothiazoline-6-sulfonic acid (ABTS^+^) was purchased from Shanghai Maclin Biochemical Technology Co., Ltd. (Shanghai, China).

Experimental apparatus: A DHT-450A high-temperature blast drying oven (Shanghai Daohan Industrial Co., Ltd., Shanghai, China), A 2500C grinder (Yongkang Hongsun Electromechanical Co., Ltd., Zhejiang, China), SQP electronic balance (Sartorius Scientific Instruments, Beijing, China), KQ-500DE ultrasonic extractor (Kunshan Ultrasonic Instrument Co., Ltd. Jiangsu, China), HHS-21-6 thermostat water bath (Shanghai Boxun Industrial Co., Ltd., Medical Equipment Factory, Shanghai, China), T6 spectrophotometer (Beijing Purkay General Instrument Co. Ltd., Beijing, China), and NEO15R high-speed refrigerated centrifuge (Shanghai Lishen Scientific Instrument Co., Ltd., Shanghai, China) were the instruments used in this study.

### Method

#### Construction of the standard curve

The polyphenol content was determined by the Folinol colorimetric method [29]. For this purpose, gallic acid monohydrate was used as the standard to prepare solutions with concentrations of 0, 10, 20, 30, 40 and 50 mg/L and the experiment was run with three replicates for each gradient. After measuring the absorbance value at 765 nm, the standard curve was made with gallic acid concentration as abscissa and the absorbance as ordinate. The regression equation was y = 0.0141x + 0.0134 (R^2^ = 0.999).

#### Determining the polyphenol content

The mycelium powder of *A. cinnamomea* was weighed and a proportional volume of DES was added. The sample was then subjected to ultrasonic-assisted extraction as required by the different extraction conditions and after centrifugation, the resulting supernatant was diluted as specified and the polyphenol concentration was determined. Polyphenol content was calculated using the following Eq. (2):

2 $${\text{Polyphenol}}\;{\text{content}}\;\left( {\text{mg/g}} \right) = \left( {{\text{C}}*{\text{V}}*{\text{n}}} \right)/{\text{M}}$$

where C is the mass concentration of the extract (mg/mL), V is the volume of the solution (mL), N is the dilution factor, and M is the dry weight of the sample (g).

#### Preparation of deep eutectic solvents (DESs)

DESs are made up of different hydrogen bond donors as well as different hydrogen bond receptors. In this experiment, choline chloride was selected as the hydrogen bond receptor. In order to investigate the extraction efficiency of polyphenols in *A. cinnamomea*, the suitability of different hydrogen bond donors was explored. Choline chloride and each hydrogen bond donor were weighed in a certain molar ratio and added to a round-bottom flask. The reagents were then heated in a water bath at 80–90 °C for 4–6 h while stirring continuously until a colorless transparent liquid, the DES, was formed. The viscosity of the eutectic solvent increases with decreasing temperature. Therefore, a certain proportion of water was added to reduce the viscosity of DESs. Since DESs have stable properties, they were stored directly at room temperature. In this experiment, different types of deep eutectic solvents were synthesized as shown in Table 3.

**Table 3:** Composition of the deep eutectic solvents

Number	Composition	Molar ratio
DES-1	Choline chloride: glucose	1:1
DES-2	Choline chloride: lactic acid	1:1
DES-3	Choline chloride: glycerol	1:1
DES-4	Choline chloride: 1,4-Butanediol	1:5
DES-5	Choline chloride: malic acid	1:1
DES-6	Choline chloride: urea	1:2
DES-7	Choline chloride: urea: glycerol	1:1:1
DES-8	Choline chloride: malonic acid	1:1

#### Effect of water content of ethanol on the extraction yield of polyphenols.

With a solid–liquid of 50 mg/mL, the effects of 30%, 50%, 70%, 90%, and 100% of ethanol on the extraction amounts of polyphenols were compared. For accuracy, each group of experiments was repeated three times, and the optimal water content of ethanol was determined by comparing the polyphenols content.

#### Effects of different formulae of deep eutectic solvents on the extraction efficiency of polyphenols from *A. cinnamomea.*

The mycelium powder was accurately weighed to achieve concentrations of 50 mg/mL. Due to the viscosity of DES, different types of DESs with a moisture content of 40% were used. In order to better reflect the superiority of deep eutectic solvent, ethanol, at a concentration of 70%, was used as a control. In the ultrasonic extractor, the extraction power was 200 W, the extraction temperature was 50 °C, and the extraction time was 30 min. Each group of experiments was repeated three times. The optimal combination was found to be DES-8 by comparing the resulting polyphenols content.

### Single factor experiment for ultrasonic-assisted extraction of polyphenols from *A. cinnamomea.*

#### Effect of solid–liquid ratio on the extraction yield of polyphenols

The mycelium powder was accurately weighed to achieve concentrations of 10 mg/mL, 20 mg/mL, 30 mg/mL, 40 mg/mL and 50 mg/mL, in DES-8, with a water content of 40%, was added. The extraction was carried out with an ultrasonic extractor at a power of 200 W, an extraction temperature of 50 °C, and an extraction time of 30 min. For accuracy, each group of experiments was repeated three times, and the optimal solid–liquid ratio was determined by comparing the polyphenols content.

#### Effect of the extraction temperature on the extraction yield of polyphenols

For a solid–liquid of 50 mg/mL, the mycelium powder of *A. cinnamomea* was accurately weighed, and DES-8, with a water content of 40%, was added. The extraction process was carried out as before except for the extraction temperatures which were 30 °C, 40 °C, 50 °C, 60 °C, and 70 °C.

#### Effect of the extraction time on the extraction yield of polyphenols

With a solid–liquid 50 mg/mL containing the mycelium powder and DES-8 with a water content of 40%, the extraction process was carried out as before except for the extraction time which were 30 min, 50 min, 70 min, 90 min, and 120 min**.**

#### Effect of water content of deep eutectic solvent on the extraction yield of polyphenols

The mycelium powder of *A. cinnamomea* was accurately weighed to achieve concentrations of 50 mg/mL. The extraction process was then carried out as described before except for the DES -8 with a water content of 0%, 10%, 20%, 30%, 40% was then added.

### Optimization of the extraction process of polyphenols using response surface methodology

Based on the results of the single factor experiment, four main influencing factors namely the ratio of the material liquid, the water content of DES-8, the extraction temperature and the extraction time were selected to optimize the experiment.

Combined with the box-Behnken experimental principle in the response surface curve method, the design-Expert10 software was used to conduct response surface analysis at the 4 factors and 3 levels (Table 4). Each factor was designed at low, medium and high levels and marked as − 1, 0 and 1 respectively.

**Table 4:** Factors and levels in response surface analysis

Factors	Levels		
	− 1	0	1
A. Ratio of the material liquid (mg/mL)	10	30	50
B. The water content (%)	0	20	40
C. Extraction temperature (°C)	40	60	80
D. Extraction time (%)	40	70	100

### In vitro antioxidant activity of polyphenols from *A. cinnamomea*

#### DPPH scavenging activity of polyphenols from *A. cinnamomea*

The DPPH scavenging experiment was performed according to the method of Xican Li [30], with slight modifications. VC was used as the control. A 0.004% DPPH-ethanol solution was prepared and stored in a refrigerator at 4 °C until used. To 1 mL of sample solution at different concentrations (0.005 mg/mL, 0.0067 mg/mL, 0.01 mg/mL, 0.02 mg/mL, 0.1 mg/mL and 0.25 mg/mL), 1 mL of 0.004% of DPPH solution and 2 mL of anhydrous ethanol were added. The reaction was allowed to progress by keeping the test tubes undisturbed in the dark for 30 min. The absorbance values were subsequently measured at 517 nm and marked as A_x_. Distilled water was used as blank and marked as A_0_. The control group was set as a sample, which was marked as A_y_. The scavenging activity was calculated using the following Eq. (3):

3 $${\text{Scavenging}}\;{\text{activity}}\;\left( \% \right) = \left[ {{\text{A}}_{0} - \left( {{\text{A}}_{{\text{x}}} - {\text{A}}_{{\text{y}}} } \right)} \right]/{\text{A}}_{0} \times {1}00\%$$

#### ABTS^+^ scavenging activity of polyphenols from ***A. cinnamomea***

The ABTS^+^ **s**cavenging experiment was slightly modified from the method of Miller [31]. VC was used as the control. ABTS^+^ (7.4 mmol/L) was first mixed with 2.6 mmol/L of K_2_S_2_O_8_ in a ratio of 1:1, and the dark light reaction was performed at room temperature for 12 h. After 40–50 times dilution, the absorbance was measured at 734 nm. The absorbance was controlled within 0.7 ± 0.02 to obtain the ABTS^+^ working solution. To 1 mL of sample solutions of different concentrations (0.005 mg/mL, 0.0067 mg/mL, 0.01 mg/mL, 0.02 mg/mL, 0.1 mg/mL and 0.25 mg/mL), 4 mL of ABTS^+^ working solution was added. After shaking well, the mixture was allowed to stand for 6 min, before reading the absorbance at 734 nm, with the values marked as A. Anhydrous ethanol was used as blank and marked as A_0_. The scavenging activity was calculated using the following Eq. (4):

4 $${\text{Scavenging}}\;{\text{activity}}\;\left( \% \right) = \left( {{\text{A}}_{0} - {\text{A}}} \right)/{\text{A}}_{0} \times {1}00\%$$

#### · OH scavenging activity of polyphenols from ***A. cinnamomea***

The scavenging capacity of polyphenols on ·OH, with VC used as a control, was determined using the H_2_O_2_/Fe system, with slight modifications of the method of Smirnoff [32]. Solutions of 9 mmol/L of FeSO_4_ and 9 mmol/L of salicylic acid–ethanol were first prepared for later use. 250 µL of 30% H_2_O_2_ was then diluted with water to 250 mL and stored in a brown reagent bottle for later use. To 1 mL of ferric sulfate, 2 mL of salicylic acid- ethanol solution was added, before adding 1 mL of sample solution of different concentrations (0.005 mg/mL, 0.0067 mg/mL, 0.01 mg/mL, 0.02 mg/mL, 0.1 mg/mL and 0.25 mg/mL) to the test tube. After mixing, 2 mL of hydrogen peroxide was added and quickly mixed. The mixture was then kept in a water bath at 37 °C for 1 h, and the absorbance value was measured at 510 nm. Values for the samples were marked as A_x._ Results with distilled water, used as blank, were marked as A_0_, while those obtained with the control group were marked as A_y_. The scavenging activity was calculated using the following Eq. (5):

5 $${\text{Scavenging}}\;{\text{activity}}\left( \% \right) = \left[ {{\text{A}}_{0} - \left( {{\text{A}}_{{\text{x}}} - {\text{A}}_{{\text{y}}} } \right)} \right]/{\text{A}}_{0} \times {1}00\%$$

#### Statistical analysis

All experiments were reproduced at least three times, with the results from single factor experiments presented as the mean ± standard deviation (SD) prior to being analyzed using SPSS (IBM SPSS Statistics 26) software. Statistically significant differences between means were then determined by one-way ANOVA, followed by Tukey's post-hoc test, with *P*-values < 0.05 considered as statistically significant. In addition, IC_50_ values for antioxidant assays—the concentration of extracts that was required to scavenge 50% of radicals—were calculated.

## Data Availability

Samples of the compounds of *A. cinnamomea* are available from the authors. And All data generated or analysed during this study are included in this published article.
